# Dithiocarbamates: Challenges, Control, and Approaches to Excellent Yield, Characterization, and Their Biological Applications

**DOI:** 10.1155/2019/8260496

**Published:** 2019-02-06

**Authors:** Ayodele T. Odularu, Peter A. Ajibade

**Affiliations:** ^1^Department of Chemistry, University of Fort Hare, Private Bag X1314, Alice 5700, South Africa; ^2^School of Chemistry and Physics, University of KwaZulu-Natal, Pietermaritzburg Campus, Scottsville 3209, South Africa

## Abstract

Progresses made in previous researches on syntheses of dithiocarbamates led to increase in further researches. This paper reviews concisely the challenges experienced during the synthesis of dithiocarbamate and mechanisms to overcome them in order to obtain accurate results. Aspects of its precursor's uses to synthesize adducts, nanoparticles, and nanocomposites are reported. Some common characterization techniques used for the synthesized products were assessed. Biological applications are also reported.

## 1. Introduction

### 1.1. Dithiocarbamates

Dithiocarbamates (dtcs) are organosulfur ligands which form stable complexes with metals [1]. The two types of dithiocarbamates are mono- and dialkyl-dithiocarbamates. The two are formed depending on the nature of amines used during the synthesis of the compound [2].

Chemistry of dithiocarbamates could be dated to start in the early twentieth century [3], precisely, in 1930 [1]. Commercial application was used as a fungicide for the first time during World War II [4]. Other wide applications can be seen in the fields of accelerating vulcanization, acting as flotation agents, agriculture (pesticide), biology, materials science, medicine, organic synthesis, photo-stabilizing polymers, and protecting radiators [5, 6].

### 1.2. Synthesis of Dithiocarbamates

Dithiocarbamates are compounds formed from the reaction of either a primary (equation (1)) or secondary amines (equation (2)) with cold carbon(IV) sulfide in basic media [2] or alcoholic solution [7]. The basic medium can either be a weak base (ammonia solution) or strong bases (sodium hydroxide and potassium hydroxide). Bases are incorporated to conserve the amines [8]. General method of preparation is called one-pot synthesis, where the synthesis reactions can be seen in equations (1) and (2) [9]. The two approaches to synthesis are the insertion reaction approach and replacement (substitution) reaction approach [10–12]. The better of the two approaches is replacement because it gives dithiocarbamates compounds of high purity [10]. Recently, Aly et al. reported the synthesis of dithiocarbamate at room temperature against the usual cold less than four degrees Celsius temperature [13]:

(1) $$\begin{array}{c} {\text{RNH}}_{2\left(\text{l}/\text{aq}\right)}+{\text{CS}}_{2\left(\text{l}\right)}+{\text{BOH}}_{\left(\text{aq}\right)}⟶{\text{RHNCS}}_{2}{\text{B}}_{\left(\text{l}/\text{s}\right)}+{\text{H}}_{2}{\text{O}}_{\left(\text{l}\right)}\;\left(\text{B}:{{\text{NH}}_{4}}^{+},\text{ Na},\mathrm{or}\text{ K}\right) \end{array}$$

(2) $$\begin{array}{c} {\text{R}}_{2}{\text{NH}}_{\left(\text{l}/\text{aq}\right)}+{\text{CS}}_{2\left(\text{l}\right)}+{\text{BOH}}_{\left(\text{aq}\right)}⟶{\text{R}}_{2}{\text{NCS}}_{2}{\text{B}}_{\left(\text{l}/\text{s}\right)}+{\text{H}}_{2}{\text{O}}_{\left(\text{l}\right)}\;\left(\text{B}:{{\text{NH}}_{4}}^{+},\text{ Na},\mathrm{or}\text{ K}\right) \end{array}$$

## 2. Challenges Encountered during the Synthesis and How to Overcome Them

First of all, synthesis must always be done in a tidy environment. Dithiocarbamates are easy to prepare and at the same time need caution during their syntheses. All glassware to be used for the syntheses must be very clean by washing with dilute trioxonitrate(V) acid if very dirty or soapy solution if less dirty. They are washed with tap water, rinsed with acetone, washed with tap water again, and finally rinsed with a lot of distilled water before properly dried in the oven. If glassware is not clean, there might be possibility of delay in the growth of crystals of the compound and might lead to error in result of elemental analysis. When the materials are ready, the experiment is set up, with a magnetic stirrer, magnetic bar stirrer, a reaction vessel, and a clean dried Erlenmeyer flask, all in the fume cupboard. The magnetic bar must be cleaned using acetone solvent before use, if possible should be soaked in the solvent overnight prior to the day of synthesis. Once it is removed, it should be rinsed with distilled water, wiped carefully with a paper towel, and put in an Erlenmeyer flask. The Erlenmeyer flask containing the magnetic bar stirrer is placed in the reaction vessel, where ice blocks or ice chips are put to fill the reaction vessel to the brim (ice bath), since the reaction in most cases takes place at a cold temperature. This temperature helps in the precipitation of the dithiocarbamate to give solid products. The magnetic stirrer, where the reaction vessel is placed on, is plugged to a switch and turned on. The amine of choice is put first in the Erlenmeyer flask, followed by the base, and finally, the refrigerated carbon(IV) sulfide is added after a while. The solution is made to stir for a specific duration based on the colour formed. Colours associated with complete synthesis are pink, yellow, yellowish-red, and white.

### 2.1. Order of Addition of Reagents for Synthesis of Dithiocarbamates

Reactants for the synthesis of dithiocarbamates are amines, base, and carbon(IV) sulfide. The order of addition of reagent has no influence on the product formed, provided, they are stoichiometrically right. Researchers like Ayyavoo et al., Ramos et al., Ramos-Espinosa et al., Sha et al., and Tarique added the amine to the Erlenmeyer flask as the first reagent, secondly the base, and thirdly the carbon disulfide [2, 3, 7, 9, 13], while Al-Mukhtar and Aghwan added the base first, followed by the amine, and lastly the carbon disulfide [14]. All reagents are added dropwise to prevent oligomerization, unwanted side products, vigorous reaction and to control the temperature, as well as, increase selectivity. In the case of the amines, solid amine first undergoes solubility test to determine the right solvent which dissolves the solid. A solution of this will be used during synthesis. On the contrary, the liquid amine can be used directly.

### 2.2. States of Product

There are two states which are generally formed for dithiocarbamates. They are either in the liquid state or solid state. In other words, in situations where solid products are not formed, a free solution or viscous solution is formed. For the liquid state, the solution is filtered. The filtrate in a clean labelled vessel is either put in the fume cupboard for slow evaporation to generate crystals or refrigerated depending on the condition which suits the growth. Another form of liquid in which a product of dithiocarbamate can exist is in the form of viscous oil. Characterization techniques can be performed directly on viscous dithiocarbamate. The other state is the solid form, known as precipitate. Precipitate can be washed, dried, and weighed for further applications, such as coordination compounds' preparation and materials chemistry. Crystals can be grown from both states of dithiocarbamates.

## 3. Washing, Drying, and Packing of Synthesized Product of Dithiocarbamate

### 3.1. Washing of Synthesized Product of Dithiocarbamate

Solid samples usually undergo washing by filtration. The different forms of filtrations are the paper filtration and filtration by suction. The better option is the filtration by suction where the precipitate is washed thoroughly by solvent. Both forms of filtration demand filter papers. In most cases, diethyl ether or cold ethanol are common solvents used for washing. The aim of washing is to remove impurities (unreacted reactants and by-products) from the desired product. The colour of the washed product is either white or cream on the filter paper.

### 3.2. Drying of Synthesized Product of Dithiocarbamate and Overcoming Challenge of Air- and Temperature-Sensitive Ammonium Dithiocarbamate

Dried dithiocarbamate can either be stored in the refrigerator for the nonstable air- and moisture-sensitive dithiocarbamate at room temperature or in the desiccator for the stable dithiocarbamate. Another approach is the use of infrared lamp.

#### 3.2.1. Air- and Moisture-Sensitive Dithiocarbamate at Room Temperature Stored in Refrigerator

The nonstable air- and temperature-sensitive dithiocarbamates are ammonium dithiocarbamates. The nonstability might be due to strength of ammonia solution used as a weak base. In order to overcome the challenge of air- and temperature-sensitive ammonium dithiocarbamate, the washed dithiocarbamate is left in a clean vessel stored in a refrigerator and removed when needed for characterization.

#### 3.2.2. Stable Dithiocarbamates

Strong bases such as sodium hydroxide and potassium hydroxide form stable products of sodium salt of dithiocarbamate and potassium salt of dithiocarbamate, respectively. Dithiocarbamate is dried mostly in a desiccator containing silica gel. Other desiccants are calcium hydroxide, calcium trioxocarbonate, and sodium hydroxide, but silica gel is preferred better so as to prevent contamination with the product (dithiocarbamate). The silica gel must be blue to indicate its active and useful states before pouring in the desiccator. Black silica gel shows that the silica gel can no longer be used and can be discarded, while red silica gel signifies requirement for it to be charged by heating in an oven. Dithiocarbamate remains inside the desiccator to use at appropriate period. Caution is to be exercised in the use of filter paper by ensuring proper drying of sample in the desiccator so as not to scratch the paper when packing. Scratching introduces more carbon into the sample, and the percentage composition of the carbon in the result for elemental analysis becomes higher than the expected value.

#### 3.2.3. Infrared (IR) Lamp

Infrared lamp avoids the use of filter paper and dries the samples for instant packing [15].

### 3.3. Packing of Dithiocarbamate after Drying

From filter papers, products are first checked if properly dried before packing. How? The appearance is free from the filter paper, but if not properly dried, it sticks to the filter paper. Products in lumps are to be broken down with clean spatula to fine particles and left inside the desiccator for proper drying. Another way is to use anhydrous copper(II) tetraoxosulphate(VI) for crude testing. The properly dried products of dithiocarbamates are packed in sample bottles and labelled for identification.

## 4. Functional Group (Moiety) of Dithiocarbamates

**Figure 1:**
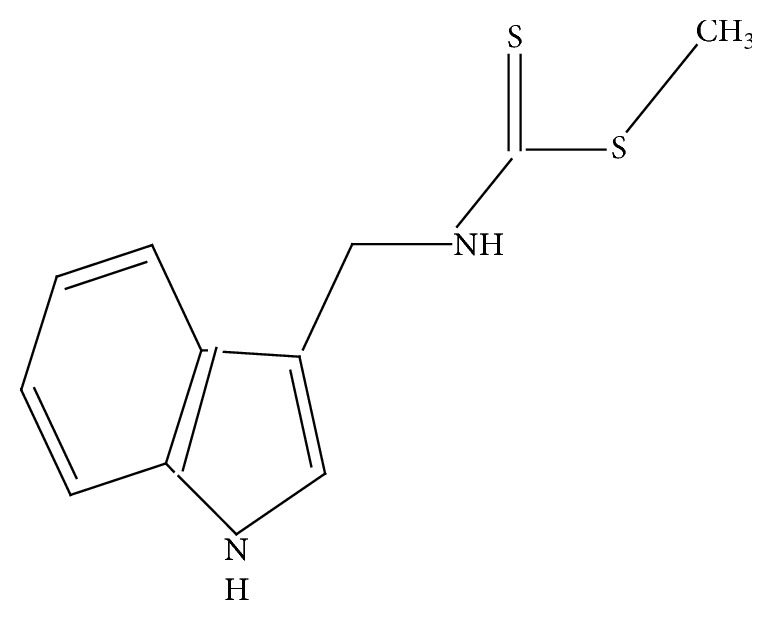
Chemical structure of brassinin.

Dithiocarbamates are compounds with the functional group called the dithiocarbamato group. They have a general formula of N-CS_2_. The International Union of Pure and Applied Chemistry (IUPAC) name for dithiocarbamates is called carbamodithioates. They are interesting organic compounds because of their phytoalexin called brassinin, which was first isolated from cabbage [16]. Brassinin can be extracted from broccoli, cauliflower, and lettuce because of their chempreventive and anticancer activities [8]. The IUPAC name for brassinin is methyl N-(1H-indol-3-ylmethyl) carbamodithioate. Brassinin has anticancer activity against human acute thymphoblastic leukemia cells. Brassinin and its derivatives inhibit indolamine 2, 3-deoxygenase (IDO), a new cancer immunosuppression objective [16]. The chemical structure of brassinin is shown in Figure 1.

### 4.1. Anomalous Nature of Dithiocarbamates

Dithiocarbamates are termed anomalous because under mild conditions, crystals of thiourea could be formed by self-condensation [17]. In this case, the two sulfur atoms are reduced to one sulfur atom:

(3) $$\begin{array}{c} \text{ammonium dithiocarbamate}+\text{heat condition}⟶\text{thiourea} \end{array}$$

## 5. Chelating Property and Coordination Compounds

Kanchi et al. reported the ability of dithiocarbamates to act as strong chelating agents towards metals to give coordination compounds which have various applications [8]. In support of this, Kamaludin et al. and Tarique et al. termed dithiocarbamates as versatile ligands because they have the affinities to chelate with various types of metals [12, 18]. The chelating abilities are due to their possession of two donor sulfur atoms in the ligands. The general formula is (*R*_1_*R*_2_) N-(C=S)SX, where *R* can be substituted by an alkyl, alkylene, aryl, or other groups, and *X* by a metal ion.

**Figure 2:**
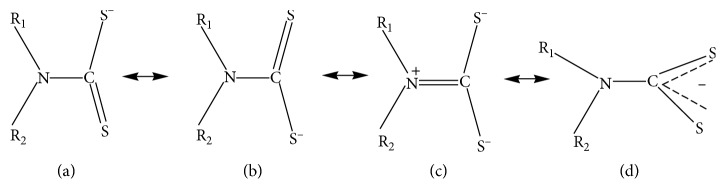
The four resonance forms of dithiocarbamates.

On the other side, Hogarth described dithiocarbamates as planar sterical ligands which are able to be modified electronically by choice of ligands [1]. Dithiocarbamates stabilize metals of different oxidation states because of the existence of soft dithiocarbamates and hard thioureide resonance forms (Figure 2). This resonance form (iii) is the outcome from delocalization of the nitrogen lone pairs onto the sulfur atom. Kaila et al. also stated another reason that the presence of a small bite angle of the of dithiocarbamate moiety contributed to the unique property as stabilizing chelating ligand [19].

### 5.1. Synthesis (Preparation) of Metal Dithiocarbamate Coordinate Compounds

The main group or transition metals dithiocarbamate coordinate compounds (metal dithiocarbamate complexes) can either be synthesized with the addition of aqueous solution of metallic salt to dithiocarbamate ligand solution [20] or the addition of dithiocarbamate ligand solution to aqueous solution of metallic salt [21–23]:

(4) $$\begin{array}{c} {2\text{RHNCS}}_{2}{\text{B}}_{\left(\text{aq}\right)}+{\text{MX}}_{2\left(\text{aq}\right)}⟶\text{M}{\left({\text{RHNCS}}_{2}\right)}_{2\left(\text{l}/\text{s}\right)}+{2\text{BX}}_{\left(\text{l}\right)}\;\left(\text{B}:{\text{Na}}^{+}\text{ or }{\text{K}}^{+};\;\;{\text{MX}}_{2}:\text{metallic salt of transition diatomic elements};\text{R can be aliphatic or aromatic substituent}\right) \end{array}$$

(5) $$\begin{array}{c} {2\text{R}}_{2}{\text{NCS}}_{2}{\text{B}}_{\left(\text{aq}\right)}+{\text{MX}}_{2\left(\text{aq}\right)}⟶\text{M}{\left({\text{R}}_{2}{\text{NCS}}_{2}\right)}_{2\left(\text{l}/\text{s}\right)}+{2\text{BX}}_{\left(\text{l}\right)}\;\left(\text{B}:{\text{Na}}^{+}\text{ or }{\text{K}}^{+};\;\;{\text{MX}}_{2}:\text{metallic salt of transition divalent elements};\text{R can be aliphatic or aromatic substituent}\right) \end{array}$$

**Figure 3:**
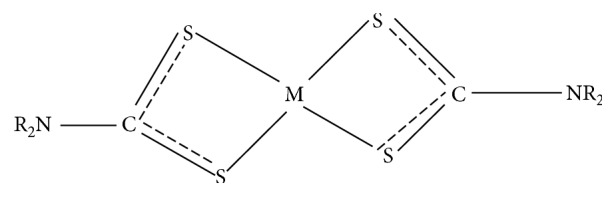
Bidentate symmetrical bonding.

**Figure 4:**
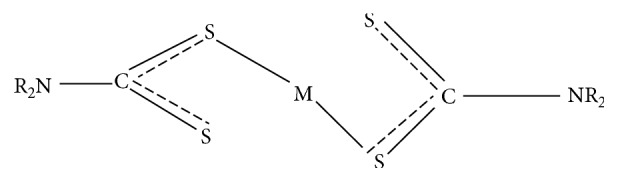
Monodentate unsymmetrical bonding.

Homoleptic complexes formed by bidentate symmetrical bonding (Figure 3) occur when the two identical dithiocarbamate ligands are used to coordinate the divalent ion [24–33]. On the contrary, heteroleptic complexes are formed by monodentate unsymmetrical binding, where another ligand is introduced with the dithiocarbamate [34, 35] (Figure 4). Rani et al. stated that the dithiocarbamate central complex, MS_2_CNR_2_, where *M* represents a metal and *R* represents an alkyl group, could prove to be of abundant synthetic uses because an extensive variety of organic substituents can be combined in this unchanging and stable bidentate structure [34]. In order to further explore the potentials of dithiocarbamates, adducts of dithiocarbamates and the metal nanoparticles are also reported.

#### 5.1.1. Adducts of Metal Dithiocarbamates Coordination Compounds

According to Al-Mukhtar et al. and Sathiyaraj et al., dithiocarbamate coordination compounds of divalent transition metals, where the central atom is partly coordinated, have the ability to reverse link molecules of organic sulfur-nitrogen-phosphorus donor bases and form intermolecular heteroleptic coordination compounds called *adducts* [33, 36]. Adducts are compounds with higher coordination numbers due to the addition of one or two Lewis bases molecules or polymerization of divalent metal dithiocarbamate complexes [33]. Lewis bases with bidentate N, N-donor bases, such as 2, 2′-bipyridine, 1, 10-phenanthroline, pyridine, methyl pyridine, and triphenylphosphine had been reported [37–45].

**Scheme 1:**
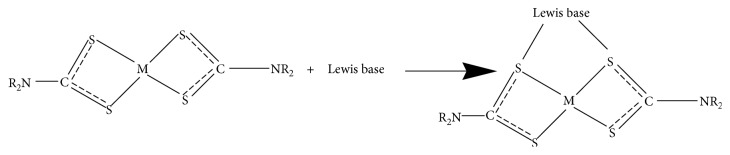
Adduct formation from bidentate dithiocarbamate and one Lewis base molecule.

**Scheme 2:**
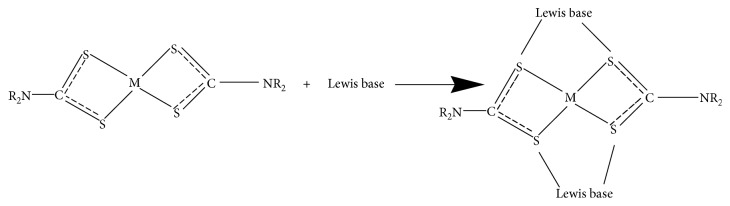
Adduct formation from bidentate dithiocarbamate and two Lewis base molecules.

On a similar note, Ekennia et al. stated that adducts formation were the interaction of metal complexes with several coordinating Lewis bases leading to retaining of the oxidation state, but an increased coordination number of metal ion in a coordination compound [46]. Obviously, the physical properties of adducts are different considerably from the parent coordination compound, which have influences on their various applications, such as photosensitizers and biological activities. Factors affecting adducts formation are coordinated ligand geometry, Lewis acid capability to accept *π* electrons, and central metal ionic size [46] (Schemes 1 and 2).

#### 5.1.2. Metal Dithiocarbamates Nanoparticles

“Nano,” means a size of 10^−9^ and measurement between 1–100 nm, which exists between two species of bulk materials and molecules. They have unique properties from these two species, partially due to the large surface to volume ratio. Nanoparticles can be amorphous, crystalline, or nanocrystals. When nanoparticles are dispersed in liquid, they form colloids with unique properties. Since many properties depend on the nanoparticles' size, monodispersity is preferred to polydispersity. Dithiocarbamate complexes are generally used as single-source precursors for metal sulfide nanoparticles because they can stabilize a wide ranging oxidation states, and secondly, the C-S bond is easy to break [47]. The set up for the nanoparticles involves a metal complex, a capping agent, and a surfactant.

*Nanoparticles Synthesis*. Top-down (physical decomposition of large materials) and bottom-up (construction from atomic and molecular precursors) are two general approaches to synthesize nanoparticles [47]. In the top-down approach, radiation- and nonradiation-based lithography methods are considered for synthesizing (fabricating) electronic nanodevices [48]. This approach is also used to fabricate nonspherical colloidal particles from a range of materials [49]. Further examples are mechanical ball milling, drawing by the mask, and uses of various plastic deformations.

**Scheme 3:**
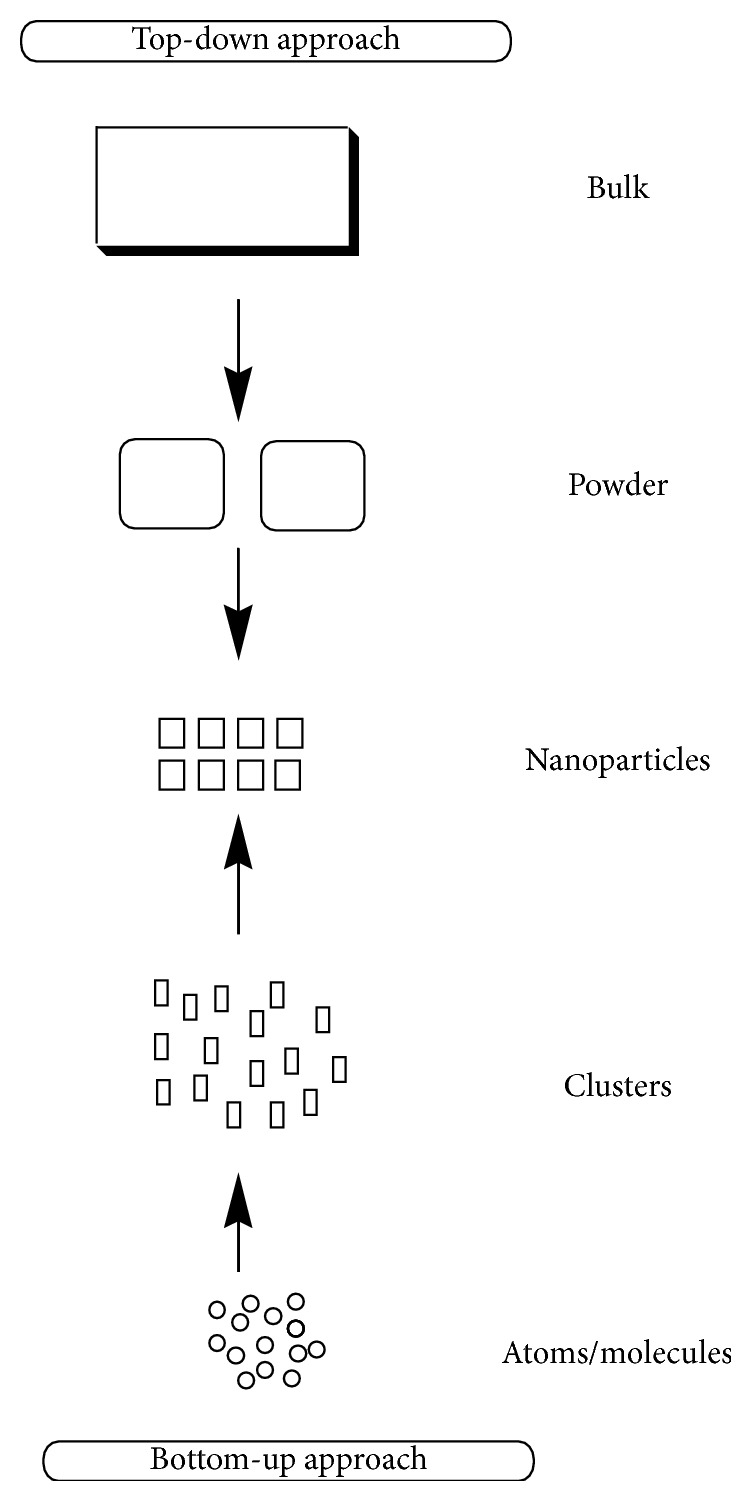
Top-down and bottom-up approaches for synthesizing nanoparticles.

On the contrary, in the bottom-up approach, the self-assembly methodology is considered to synthesize polymer nanostructures [47]. In addition, Nini et al. developed a protocol for bottom-up self-assembly of nanogaps by molecular association of gold nanoparticles (AuNPs) [50]. An oligo(phenylene-ethynylene) (OPE) molecule with two attached dithiocarbamates' groups was used to study the linking of AuNPs. The top-down and bottom-up approaches for synthesizing nanoparticles are shown in Scheme 3.

**Scheme 4:**
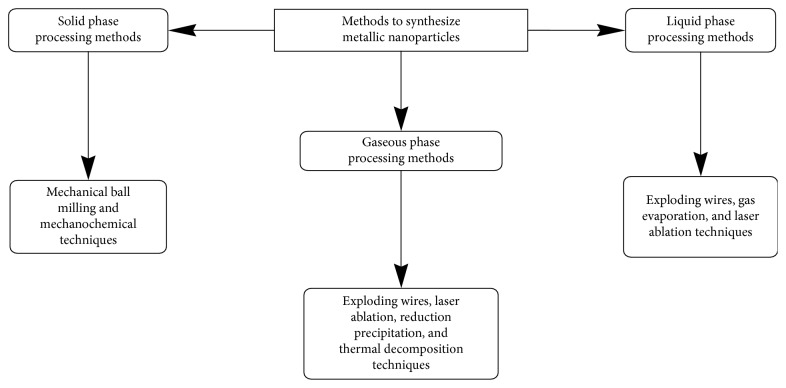
General synthetic methods to prepare metallic nanoparticles.

*Nanoparticles General Synthetic Methods and Conditions*. After characterization of metallic dithiocarbamate complexes, synthesis of metallic nanoparticles can be achieved with various methods. These methods can be solid-phase, liquid-phase, and gas-phase processes. Solid-phase methods entail mechanical ball milling and mechanochemical techniques, liquid phase methods entail exploding wires, laser ablation, reduction precipitation (colloidal methods, hydrothermal synthetic methods, sol-gel processing methods, polyol methods, and water-oil emulsions), and thermal decomposition, while, gas-phase methods entail exploding wire, gas evaporation and laser ablation techniques [51]. The general synthetic methods to prepare metallic nanoparticles is shown in Scheme 4. Inert atmosphere is used mostly as a condition to prevent surface oxidation of metal nanoparticles, whereby, nitrogen gas is used for the reaction [52]. Other conditions are ambient (air atmosphere) and room temperatures [52].

- Thermal decomposition's easy, facile, and efficient route for metallic nanoparticles has made it a choice approach compared with other methods [47, 51–64]. Sathiyaraj et al. proved the use of metal dithiocarbamate complexes as successful single-source precursors (SSPs) to prepare corresponding metallic sulfide nanoparticles [33]. From the assessment of their opinions, the synthesis of metallic dithiocarbamate complexes is to make them single-source precursors as intermediary for the synthesis of their respective metallic sulfide nanoparticles [22, 35, 64, 65]. Pawar et al. buttressed their point when they reported that thermal decomposition could be used to achieve the aim of fabricating metallic sulfide nanoparticles [66]. In addition, they further reported that SSPs have advantages over other methods, such as ligand choice which can affect volatility, limited prereactions, and low toxicity.

**Scheme 5:**
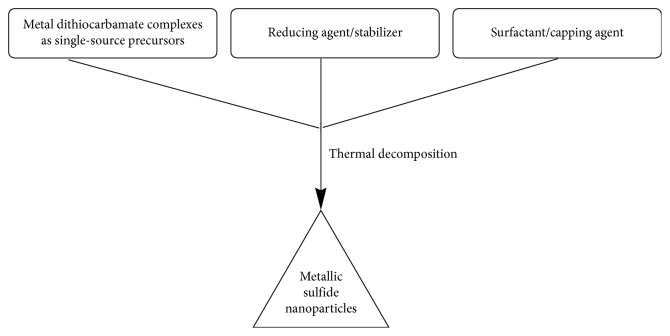
Metallic sulfide nanoparticles formed via thermal decomposition.

On a similar note, Nair and Scholes stated that the use of thermal decomposition on single-source precursors was aimed at finding out a link between the thermal decomposition and morphology (shapes) of nanoparticles obtained from the thermolysis of these precursors in a coordinating solvent [67]. Abdullah et al. and Odularu reported that the use of thermal decomposition for thermolysis of single-source precursors yields monodispersed nanoparticles, which promotes technological and biomedical applications, respectively [57, 58]. Odularu further reported the use of a reducing agent (stabilizer) to reduce the SSP to a metal, in doing so brings about stability, while the capping agent helps to prevent agglomeration [58]. Single source precursors are used alongside with stabilizers and capping agents; therefore, all are subjected to thermal decomposition. Metallic sulfide nanoparticles formed via thermal decomposition are shown in Scheme 5.

### 5.2. Nanocomposites

Nanocomposites are synthesized in order to further enhance the properties of dithiocarbamates. Henrique et al. reported nanocomposites as materials of the twenty-first century because they possess distinct design and combined properties not present in conventional composites [64]. According to Henrique et al., nanocomposites are composites with at least a phase shows dimensions in the nanometre range of 10^−9^ m [64]. In addition, they stated that nanocomposites had been developed to overcome microcomposites and monolithics' limits. Their three different categories are ceramic matrix nanocomposites (CMNC); metal matrix nanocomposites (MMNC), and polymer matric nanocomposites (PPMC) [64].

**Scheme 6:**
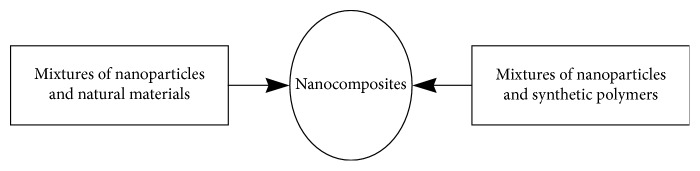
Approaches to nanocomposites synthesis.

Approaches to nanocomposites synthesis involve the dispersion of the nanoparticles in material matrices, such as natural materials and synthetic polymers (Scheme 6).

**Scheme 7:**
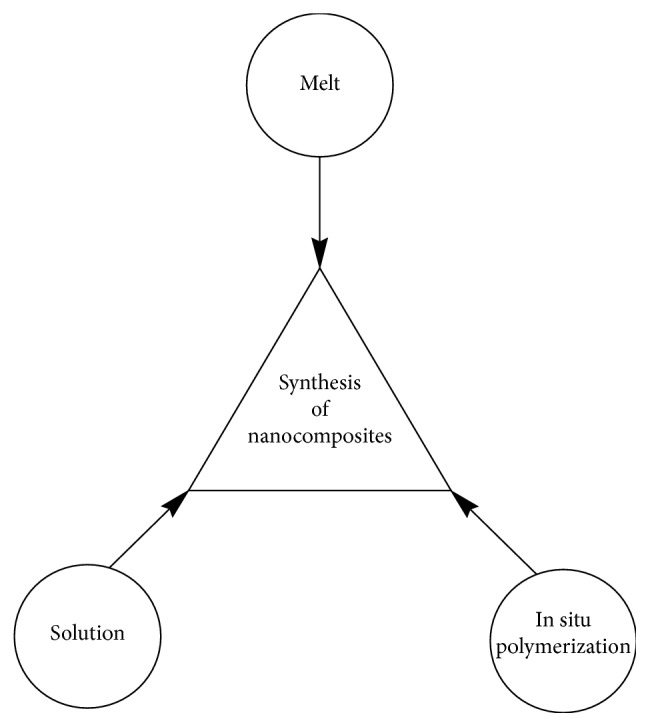
Three different ways to synthesize nanocomposites.

The approaches can be carried out in three different ways (Scheme 7), namely, melt (polymer is heated to melting point and mixed with nanoparticles), solution (polymer and nanoparticles are dissolved in right solvent and mixed together), and *in situ* polymerization (monomer and nanoparticles are mixed together to form polymer) [64, 68].

## 6. Characterization of Dithiocarbamates

The characterization techniques used for both dithiocarbamates and their coordination compounds are more of the same than similar. The characterization techniques are solubility test, physicochemical parameters (melting point and molar conductivity (MC)), elemental analysis, spectroscopy (ultraviolet-violet, infra-red, and photoluminescence), magnetic moment, and microwave spectroscopy (nuclear magnetic resonance and electron magnetic resonance). In the case of nanoparticles and nanocomposites, additional characterization techniques are needed. These are X-ray diffraction (XRD), scanning electron microscopy (SEM), transmission electron microscopy (TEM), energy-dispersive X-ray spectroscopy (EDX/EDS), and thermal analysis (TA).

### 6.1. Solubility test

Solubility is the highest concentration of a solute which can dissolve in a solvent at a certain temperature [69]. It has a unit of mol/L or g/L. Factors which influence solubility are solute's concentration, gas pressure, polarity of solute and solvent, as well as, temperature [69]. Solvents used for testing solubility are classified into polar and nonprotic solvents [70–72]. Polar solvents are further divided into polar protic (ethanol, butanol, methanol, propanol, and water) and polar aprotic solvents (1, 4-dioxan, acetone, acetonitrile, dichloromethane (DCM), dimethylformamide (DMF), dimethyl sulfoxide (DMSO), and tetrahydrofuran (THF)). Solvents termed nonpolar are benzene, chloroform, diethyl ether, ethyl acetate, hexane, and toluene [72]. Therefore, dithiocarbamate ligands of ammonium, sodium, or potassium salts are water soluble, while their corresponding coordination compounds are insoluble in water. In pharmaceutical industry, solubility of a drug is one of the conditions which justifies its acceptance as suitable for use [73]. From personal experience, the purpose of the solubility test is to check the most dissolving rates of the synthesized compounds, which would be used as guide for other characterization techniques of MC, UV-Vis, NMR, and biological studies. How possible is this? The right solvent during the solubility test, also serves as the right solvent for other characterization techniques, such as MC, UV-Vis, NMR, and biological studies.

### 6.2. Physicochemical Parameters

They involve observing the changes in appearance of the sample after undergoing chemical reactions. Two common physicochemical parameters are melting point (MP) or decomposition and molar conductivity (MC) [46, 74–76].

### 6.3. Melting Point (MP) or Decomposition

Melting point is the temperature (degree Celsius) at which a solid changes to liquid [71]. Melting point is similar to decomposition. The product obtained after a sample melts does not change in colour but in state of matter, but that of decomposition involves a change to black colour, as well as, a change in state of matter. The mercury in glass type of thermometer in a melting point apparatus is used to measure the temperature. It must be calibrated with a known mass before use. Melting, decomposition, and dissolution are separate occurrences, though they are found to overlap with one another [75]. According to Ross et al., crystalline structures of some compounds are influenced by crystalline conditions and the existence of impurities. Low melting point might be due to the existence of these impurities and deficiencies [75]. Such compounds contain noncrystalline regions which might undergo decomposition and later dissolution at the decomposition boundary and speeding up of decomposition reaction. Melting of such compounds happens as a separate thermodynamic route with no chemical change for the molecules, whereas the molecular motion of amorphous or melted areas is a precondition for decomposition [75].

Some factors which influence the melting point are arrangement and structural arrangement of the molecules, force of attraction, and presence of impurities. The aim of determining the melting point is for the identification and purity of a compound [71]. Sainorudin et al. and Amira et al. stated that compounds regarded as being pure must have their melting points less than 3°C and 2°C, respectively [71, 76].

### 6.4. Molar Conductivity (MC)

Molar conductivity is the determination of conductivity of a compound [77]. It involves the dissolution of the solid compound in the best solvent to form a test solution. The process involves the use of a molar conductivity meter. The electrode in the molar conductivity meter is first used to test three different solvents to standardize the instrument before being used for the test solution. The aim of doing molar conductivity is to test if the compound is electrolytic (ionic) or nonelectrolytic (nonionic) in nature [46, 74–76].

### 6.5. Elemental Analysis (EA)

Elemental analysis (EA) is the analysis done to check the purity of the tested compound [78]. Previous cautions were given to ensure good results for elemental analysis. Other factor to note for a good result from elemental analysis is preventing splashing of synthesized compound during its preparation. Since MP also determines purity, it differs from EA. In the case of MP, it justifies qualitative purity, while EA justifies quantitative purity. The instrument used to carry out EA is the elemental analyzer [78].

### 6.6. Spectroscopy

Spectroscopy involves the use of electromagnetic radiation on matter to reveal their molecular properties. For ligand and coordination compounds of dithiocarbamates, it involves three spectroscopic methods of ultraviolet-visible (UV-Vis) and Fourier transform infrared (FT-IR).

#### 6.6.1. Ultraviolet-Visible (UV-Vis)

It is also called electronic spectroscopy because the electronic spectra for ligands of dithiocarbamates and the coordination compounds are recorded in the UV-Vis region from a range of 200–800 nm [19]. Ligands of dithiocarbamate show three bands relative to intramolecular charge transfer in the ultraviolet region of the electromagnetic spectrum [10]. Abbas stated that “these bands are Band I which corresponds to *π*-*π*^*∗*^ transitions of the N-C=S group. Band II is assigned to *π*-*π*^*∗*^ of S-C=S group and Band III to *n*-*π*^*∗*^ transitions” [79]. In coordination compounds, the two transitions are from ligands and excitation of metal ions [71, 79]. Transitions from ligands are *π*-*π*^*∗*^ and *n*-*π*^*∗*^ while transition as a result of excitation of metal ions is called the d-d transition [71, 79]. Metal-to-ligand charge transfer (MLCT) and ligand-to-metal charge transfer (LMCT) are due to excitation of an electron from the metal ion to the dithiocarbamate ligands and vice versa [79]. This characterization technique is used to support results from other characterization techniques. The main reason for using UV-Vis spectroscopy is to determine the geometry of the proposed coordination compounds. Electronic spectra can appear as decrease in wavelength, referred to as blue shifted (hypsochromic shift), increase in wavelength, referred to as red shifted (bathochromic shift), decrease in absorptivity (hypochromic shift), and increase in absorptivity (hyperchromic shift). Absorption of ultraviolet or visible radiation agrees with excitation of outer electrons. The three types of electronic transitions are (i) transitions which consists of *σ*, *π*, and *n* electrons, (ii) transitions which consists of charge-transfer electrons, and (iii) transitions which consists of *d* and *f* electrons.

#### 6.6.2. Infrared (IR) Spectroscopy

**Figure 5:**
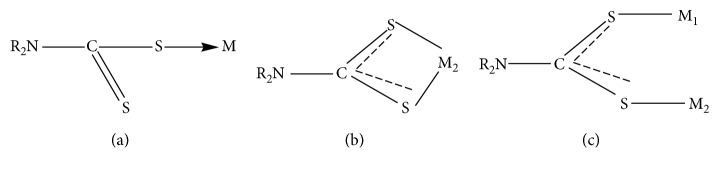
The different coordination modes of dithiocarbamates to metal(s). (a) Unidentate (monodentate). (b) Bidentate. (c) Anisobidentate bridging.

In electromagnetic radiation spectrum, unlike electronic spectroscopy where electronic transitions occur, there exist vibrations in infrared spectroscopy. There are three prominent regions in the infrared spectrum, namely, 3400–3100 cm^−1^ for ѵ_N-H_ and *v*_O−H_, 1550–1450 cm^−1^for *v*_C−N_, and 1050–950 cm^−1^ for *v* S_2_C=NR_2_ [80]. The *v* means stretching vibrations. Hogarth stated that the lipophilic nature of dithiocarbamates and their common ligation to metals gave different coordination modes of (a) unidentate (monodentate), (b) bidentate, and (c) anisobidentate bidentate bridging (Figure 5) [1]. The metal coordination is found in the far IR region of the electromagnetic spectrum, that is, in the fingerprint region.

### 6.7. Photoluminescence (PL)

Photoluminescence involves the emission of light after it had been absorbed in an optical system [26, 81–83]. Important applications of photoluminescence are to give the optical band gap in technology and biomedicine [84, 85].

### 6.8. Mass Spectroscopy (MS)

Mass spectrometry is a technique used to analyze dithiocarbamates to obtain their exact molecular masses from a mass spectrometer [86].

### 6.9. Nuclear Magnetic Resonance (NMR)

Nuclear magnetic resonance (NMR) is used as a diagnostic tool for synthesized compounds' structures. The most relevant of all nuclear magnetic resonance are proton nuclear magnetic resonance (^1^H NMR) and (^13^C NMR). The ^1^H NMR depicts the number of protons as the name decodes, as well as, the ^13^C NMR depicts the number of carbon atoms as the name decodes. The aromatic protons are known to have signals between *δ* 6 and 8 ppm, while the aromatic carbons are known to have resonance between *δ* 100 and 150 ppm [87–91]. The chemical shift of 198 ppm to 200 ≥ 220 ppm is for the dithiocarbamate group (-NCS_2_) [87–91].

### 6.10. Single-Crystal X-Ray Crystallography

Single-crystal X-ray crystallography is a technique to confirm the true and original structure of the synthesized dithiocarbamate by determining the atomic and molecular structures of the crystals. Environment suitable might be cool, dark, hot, or warm. Crystals are mostly grown in a suitable environment and subjected to the process of single-crystal X-ray crystallography. According to Kamaludin et al., the diffractometer gives the structural determination of crystals [18]. Data collected are solved and refined with relevant software.

### 6.11. X-Ray Diffraction (XRD)

Nabipour stated that X-ray Diffraction (XRD) was used to determine the composition of the nanoparticles [92]. It is sometimes referred to as the elemental analysis, but quite different from elemental analysis. The commonest application of XRD is the analysis of powders and polycrystalline materials. These analyses entail phase identification, phase quantification, amorphous content crystallinity determination, crystal structure determination with refinement, in situ crystallization analysis, Debye rings direct visualization, high-throughput screening (HTS), and measurements under nonambient conditions. An average crystalline size can be obtained using Debye–Scherrer formula: *D*=0.9*λ*/*β*cos*θ* [93]. X-ray diffraction is the elastic scattering of X-ray photons by atomic particles in a periodic lattice. Constructive interference is obtained from scattered monochromatic X-rays which are in phases. Bragg's law is used to derive lattice spacing.

Mathematically, Bragg's Law is

(6) $$\begin{array}{c} n\lambda =2d\sin\theta \left(\text{v}\right), \end{array}$$

where *n* is an integer (order of reflection), *λ* is the wavelength of X-rays, *d* is the characteristic spacing between the crystal planes of a given specimen, and *θ* is the angle between the incident beam and the normal to the reflecting lattice plane. The crystallographic phase can be determined from measuring *θ*.

### 6.12. Scanning Electron Microscopy (SEM) and Transmission Electron Microscopy (TEM)

The scanning electron microscopy (SEM) entails scattered electrons, while transmission electron microscopy (TEM) entails transmitted electrons. The SEM is applicable to sample surface and its external composition (morphology), but TEM is applicable to internal composition, such as crystallization, magnetic domains, morphology, and stress [57].

### 6.13. Sample Size, Resolution, Dimensions, and Uses

The SEM entertains analysis of large amount of samples at a time, while in TEM, samples are cut thinner, and analysis on a small amount of sample is done at a time.

The SEM has a lower resolution and provides a three-dimensional image than TEM, which has a higher resolution and provides a two-dimensional image. Resolution power of SEM is 10 nm, while TEM resolution power is 0.2 nm. Another TEM technique to measure lattice strain below the nm range is *Kikuchi lines* [94, 95]. Images of SEM are shown on monitor, while TEM images are shown on fluorescent screens.

Chemical segregations, etched microstructures, integrated circuit chips, polished microstructures, and surfaces require the use of SEM, while dislocation imaging, grain boundaries, tiny precipitates, and solid defect structures need the use of TEM.

### 6.14. Energy-Dispersive X-Ray Spectroscopy

Energy-dispersive X-ray spectroscopy is abbreviated as EDS, EDX, EDXS, or XEDS. It is described simply as Single-Crystal X-ray Diffraction (Single-Crystal XRD). The EDS is sometimes referred to energy dispersive X-ray analysis (EDXA) or energy dispersive X-ray microanalysis (EDXMA). The EDS analysis is usually done on multiple areas of the dithiocarbamate nanoparticles to confirm the presence and stoichiometry of the sample [57].

### 6.15. Thermal Analysis (TA)

Thermal analysis aims at determining the thermal stability of studied sample [57].

Thermal analysis of dithiocarbamate complexes can be studied by thermogravimetric analysis (TG/TGA), differential scanning calorimetry (DSC), and differential thermogravimetric analysis (DTA/DTG) in the presence of nitrogen or oxygen gas [57]. Thermal analysis has the capacity to provide important details concerning melting point, residue form, structural properties, and weight loss at the end of the decomposition process [96, 97]. The TGA can be done separately or done at this same time with DSC/DTA [38, 96, 97].

### 6.16. Both Thermogravimetric Analysis (TGA) and Differential Thermogravimetric analysis (DTG)

With DTG, mass loss (vapour emission) or gain (gas fixation) of samples against temperature during the heating process is obtained. Decrease in weight indicates exothermic reaction, and increase in weight means endothermic reaction. Both TGA and DTG can be obtained from the same result of temperature difference [97, 98]. Peak heights are reflections of reactivity, while smaller peak displays thermal stability.

### 6.17. Both Differential Scanning Calorimetry (DSC) and Differential Thermogravimetric Analysis (DTA)

In DTA, the dithiocarbamate sample is heated in a crucible and another reference in another crucible simultaneously [97, 98]. The DTA peak is the temperature difference between the sample and the reference, usually plotted against temperature. Both DSC and DTA are similar, but DSC, apart from temperature, also entails measurement of heat flow difference into and from the sample [97, 98]. Both DSC and DTA are used to measure compatibility, glass transition, heat capacity, melting, phase changes, polymerization, purity crystallization, purity evaporation, polymerization, pyrolysis, sublimation, etc. Results obtained from DSC are better than DTA [97, 98].

## 7. Biological Applications

Aly et al., Nabipour et al., Manav et al., and Ghorbani-Vaghei et al. identified numerous applications of dithiocarbamates in biological studies as anticancer [13], antifungal [13], antibacterial [99, 100], rodent repelling [101], growth depressing [102], and toxicity studies [18]. According to Khan et al., palladium(II) and platinum(II) coordination compounds of dithiocarbamates attracted a lot of attention due to their better anticancer, antitumour, and less toxic properties than cisplatin [103]. They further stated that the presence of nitrogen and sulfur (N and S) coordination modes in ligands used to synthesize platinum(II) complexes helped to lower the challenges linked with cisplatin's toxicity and its analogues.

Cell viability test for synthesized dithiocarbamate compounds is carried out in order to test for the safety or the cytotoxic activities in cancer cells [104]. The estimation of the lactase dehydrogenase (LDH) level is another method to study cell viability, but less sensitive than MTT assay [104]. Other methods applied to assess cell viability are based on several cellular functions, such as ATP production, cell adherence, cell membrane permeability, coenzyme production, and nucleotide acceptance activity [104]. Among all, tetrazolium (MTT) is one of the most often used, which uses colorimeter to determine cell viability. Cytotoxicity test is done to assess cell viability by a dose response compounds' toxicities [104–106]. Cytotoxicity is usually assessed with MTT [3-(4, 5-dimethylthiazol-2-yl)-2, 5-diphenyltetrazolium bromide] assay. Here, the MTT assay is a colorimetric test, where the tetrazolium salt is reduced with a colour change.

Other cytotoxicity assays are 2, 3-bis-(2-methoxy-4-nitro-sulfophenyl)-2H-Tetrazolium-5-Carboxanide (XTT), a second-generation drug which produces a water-soluble product [107], or MTS tetrazolium assay. This assay uses colorimetry to measure the reducing potential of the cell [107]. Here, viable cells will reduce the MTS reagent to a coloured formazan product. A similar electrochemical-based assay used in order to monitor viability of cells uses resazurin (a fluorescent dye). In the case of ATP assays, which entail bioluminescent assays, ATP is the limiting reaction for the luciferase reaction. Sulforhodamine B (SRB) assay, water-soluble tetrazolium salt (WST) assay, and clonogenic assay are also used to measure cytotoxicity. These assays can be combined in order to reduce assay-specific false-positive or false-negative results.

Cell viability can be obtained by taking the percentage of mean optical density (OD) results of the test samples and negative control mean of OD.

Mathematically, it is

(7) $$\begin{array}{c} \frac{\text{mean optical density }\left(\text{OD}\right)\text{ results of the test samples}}{\text{negative control mean of OD}\times 100}. \end{array}$$

For every biological activity, there is a need for controls. Controls are both positive and negative controls. Dimethyl sulfoxide is usually used as negative control, while standard drugs are used as positive controls.

## 8. Conclusion and Future Research

Synthesis of dithiocarbamate and its metal complexes can be successful with cleanliness and proper preparation. Different modifications in the areas of adducts formation and nanoparticles extended the versatility of dithiocarbamates. Relevant characterization techniques for ligands, metal complexes, and adducts formation of dithiocarbamates are melting point (decomposition), molar conductivity, elemental analysis, ultraviolet visible spectroscopy, infrared spectroscopy, mass spectroscopy, nuclear magnetic resonance, and single-crystal X-ray crystallography.

Materials chemistry involving metal dithiocarbamates nanoparticles entail further characterization techniques of SEM, TEM, EDS, and XRD. The presence of nitrogen and sulfur coordination modes has made dithiocarbamates to be successfully applied as anticancer agents. Future research will review papers on groups of aliphatic and aromatic dithiocarbamates and compare their anticancer activities with each other.
